# Supplementary material for: Segmental copy number amplifications are more stable than aneuploidies in the absence of selection
Source: Mol Biol Evol. 2026 Apr 11;43(4):msag095. doi: 10.1093/molbev/msag095 (PMC13107562; doi:10.1093/molbev/msag095)
Supplement: msag095_Supplementary_Data [file msag095_supplementary_data.zip › De_et_al_revision2_Supplementary_Figures.pdf]

# Supplementary Figures

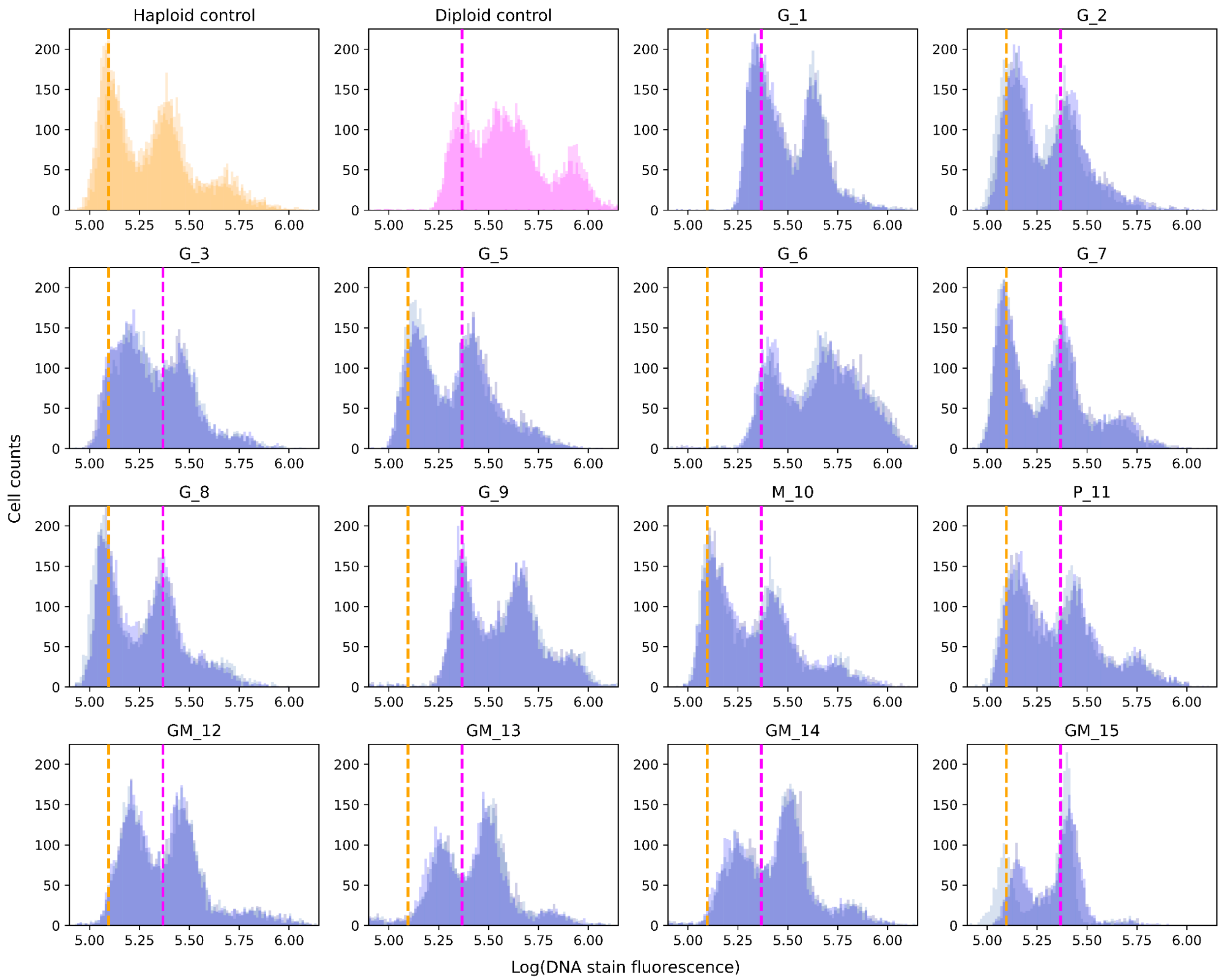


**Figure S1. Ploidy of all CNV strains used in the study.** Ploidy was measured by quantifying DNA content using propidium iodide staining of exponentially growing cells. Haploid (orange) and diploid (magenta) control strains were assayed to define ploidy states and all strains were tested in triplicate (overlaid in each subplot). Dashed lines indicate the modes calculated from all replicates of each control, and mark the position of the 1N peak for haploids (orange) and the 2N peak for diploids (magenta). All CNV strains are shown here except *G_4* (shown in Figure S2).

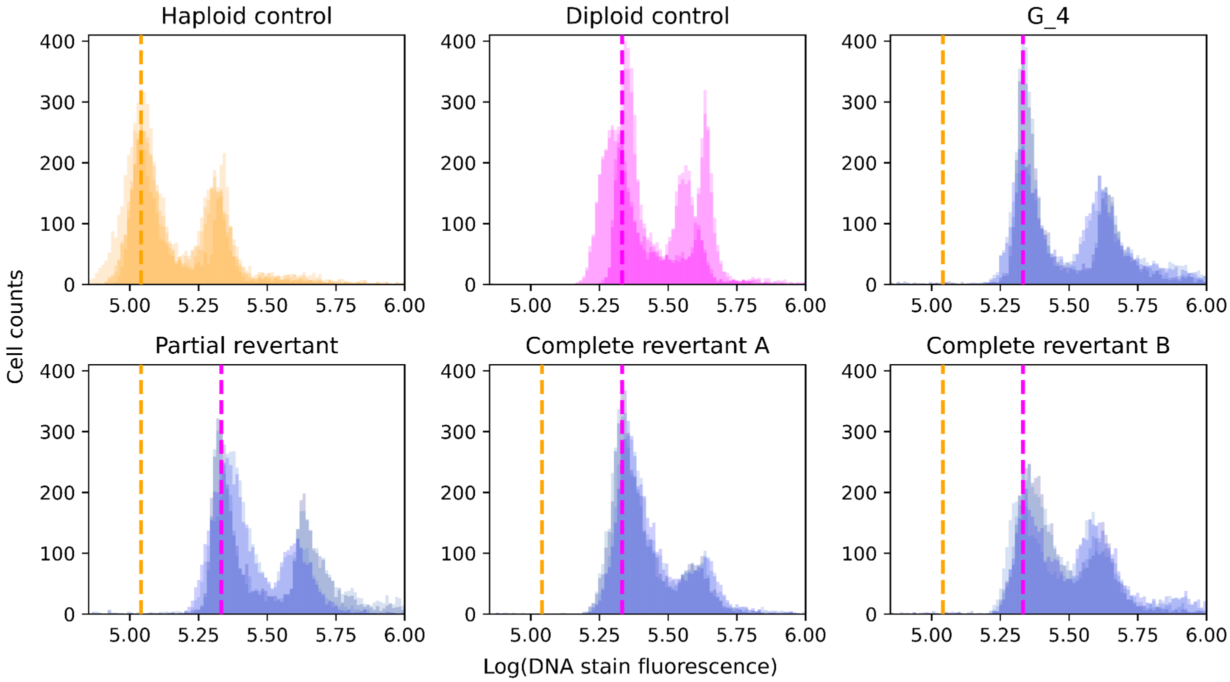


**Figure S2. Ploidy of segmental CNV strain *G_4* and derived revertants.** Ploidy was measured by quantifying DNA content using propidium iodide staining of exponentially growing cells. Haploid (orange) and diploid (magenta) control strains were assayed to define ploidy states and all strains were tested in quadruplicate (overlaid in each subplot). Dashed lines indicate the modes calculated from all replicates of each control, and mark the position of the 1N peak for haploids (orange) and the 2N peak for diploids (magenta).

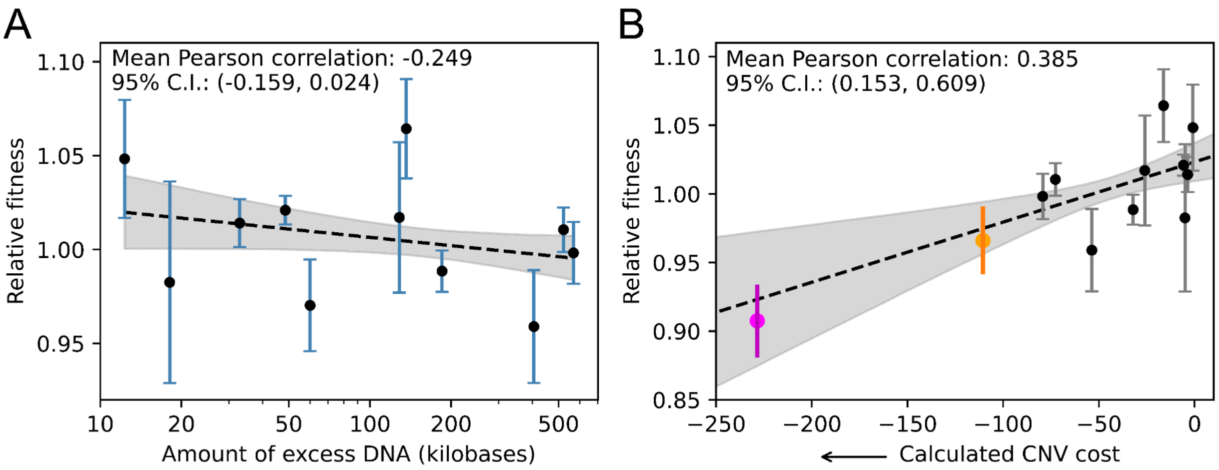


**Figure S3. Relationship of relative fitness with amount of excess DNA and with estimated CNV cost. A)** Pearson correlation coefficient was calculated between relative fitness and amount of extra DNA in all segmental CNVs. Points with error bars indicate 95% confidence intervals for relative fitness. Black dashed line and grey shadow indicate 95% confidence intervals for a linear regression through all points. Relative fitness has no significant correlation (95% CI: -0.159, 0.024) with the amount of excess DNA. **B)** Relative fitness has significant correlation (95% CI: 0.153, 0.609) with estimated fitness costs of segmental CNVs. Double aneuploidy of chromosomes XI and XIV (magenta) and single aneuploidy of chromosome XI (orange) lie on the regression line fitted to all segmental CNVs.

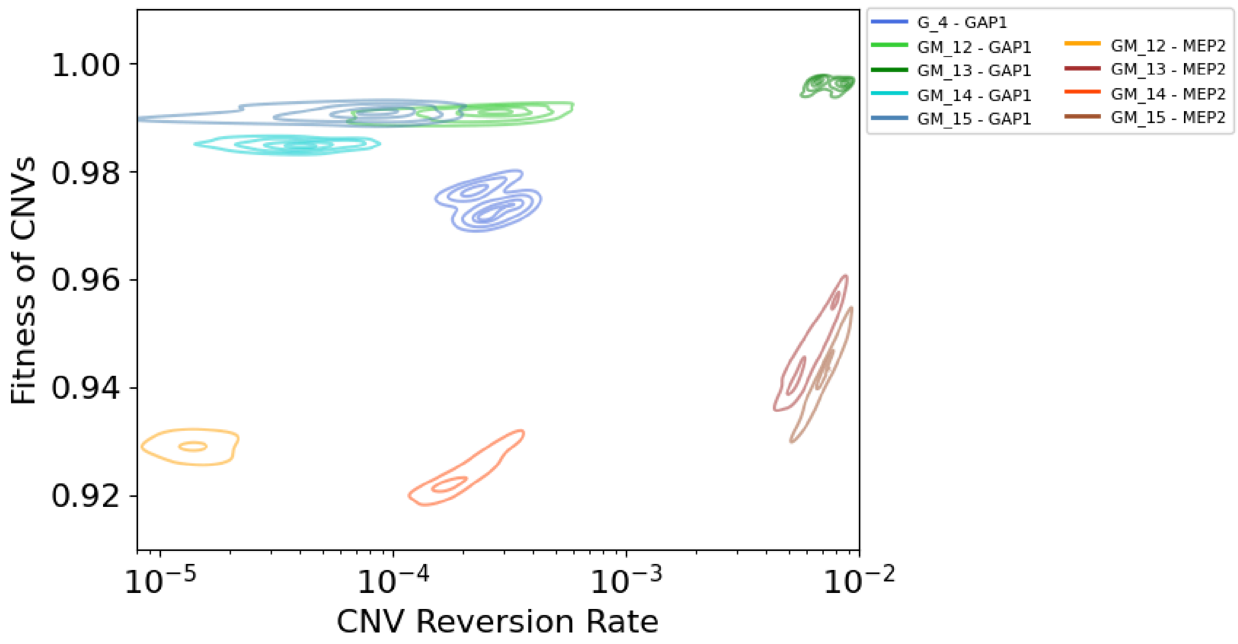


**Figure S4. Parameter estimates.** 95% high-density regions (HDRs) of the collective posterior distributions for the fitness cost (derived from selection coefficients, see Methods) and reversion rate of CNVs.

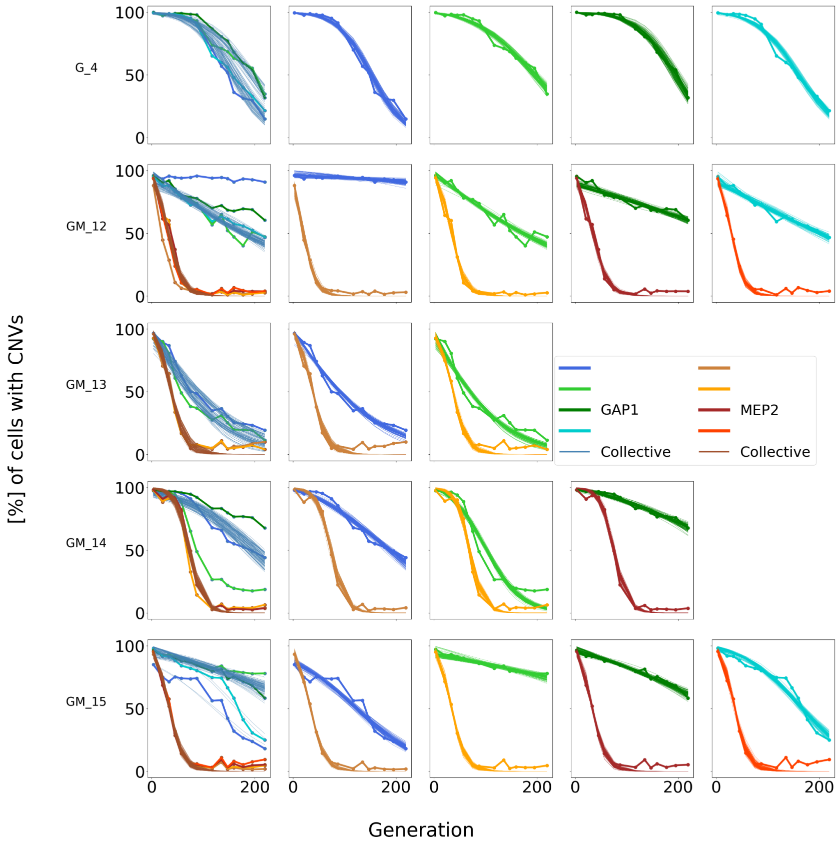


**Figure S5. Posterior predictive checks.** Model simulations of 100 parameter sets sampled from the posterior distributions inferred from each experimental replicate (shaded lines) against the empirical data (solid lines with markers). Leftmost (grey) panels of each row show predictive checks of the collective posterior distribution for the strain, i.e., a posterior distribution conditioned on all strain replicates.

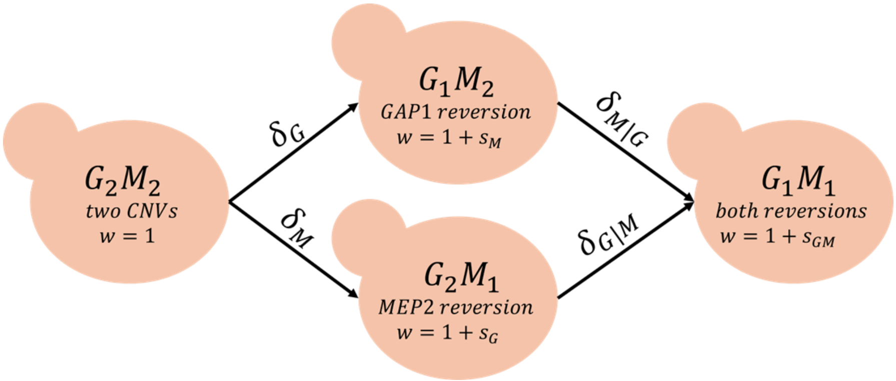


**Figure S6. A genotype-tracking evolutionary model.** CNV reversions are not necessarily independent, with rates $\delta_{i|j}$ for transitioning from genotype $j$ to genotype $i$, and a distinct selection coefficient $s_k$ for genotype $k$. We track the proportions of each genotype, from which we can calculate the total proportions of each CNV. Cell labels refer to both genes (G - *GAP1*, M - *MEP2*) and their corresponding copy numbers, i.e., indicate the entire cell's genotype.

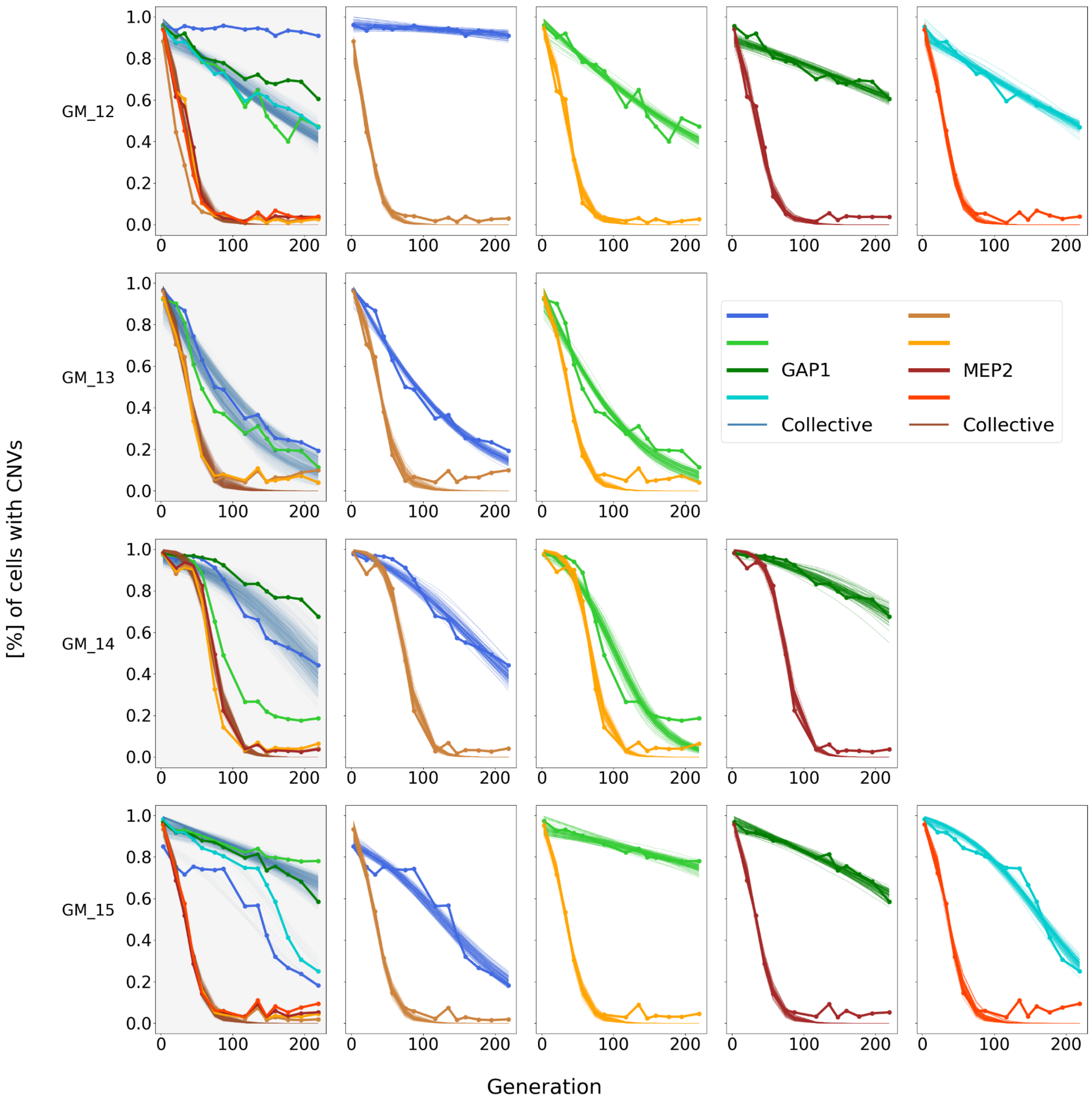


**Figure S7. Posterior predictive checks with the genotype-tracking model.** Genotype-tracking model simulations of 100 parameter sets sampled from the posterior distributions of each experimental replicate (shaded lines), inferred using the allele-tracking model against the empirical data (solid lines with markers). Leftmost (grey) panels of each row show predictive checks of the collective posterior distribution for the strain, i.e., a posterior distribution conditioned on all strain replicates. Strain *G_4* is not shown since it does not have a *MEP2* CNV.

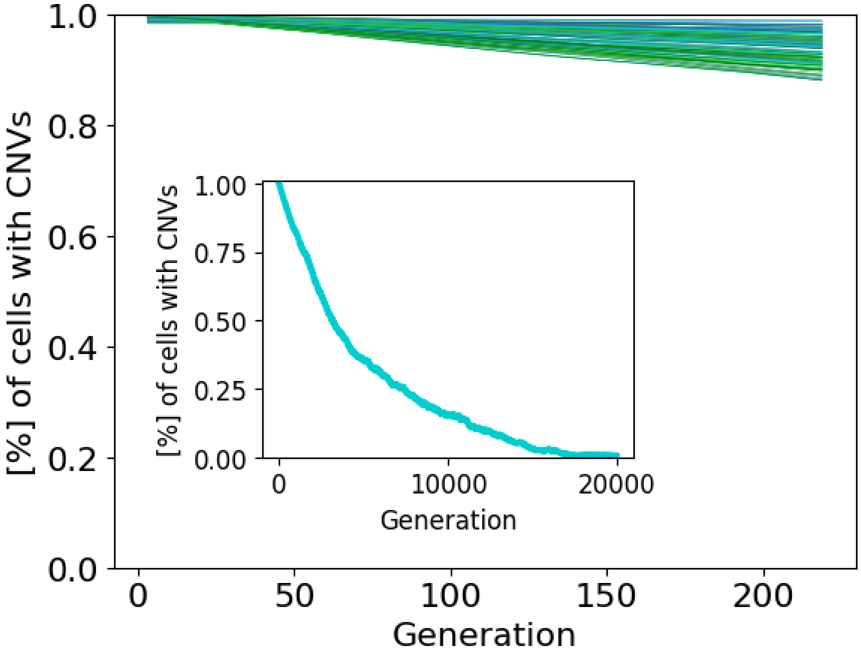


**Figure S8.** ***GAP1*** **CNV reversion without selection.** Evolutionary simulations with mutation rate and initial CNV proportion sampled from the collective posterior distribution of strain *G_4* and with very weak selection, $s = 10^{-10}$. Inset axis shows a simulation of the sample mean for 20,000 generations.

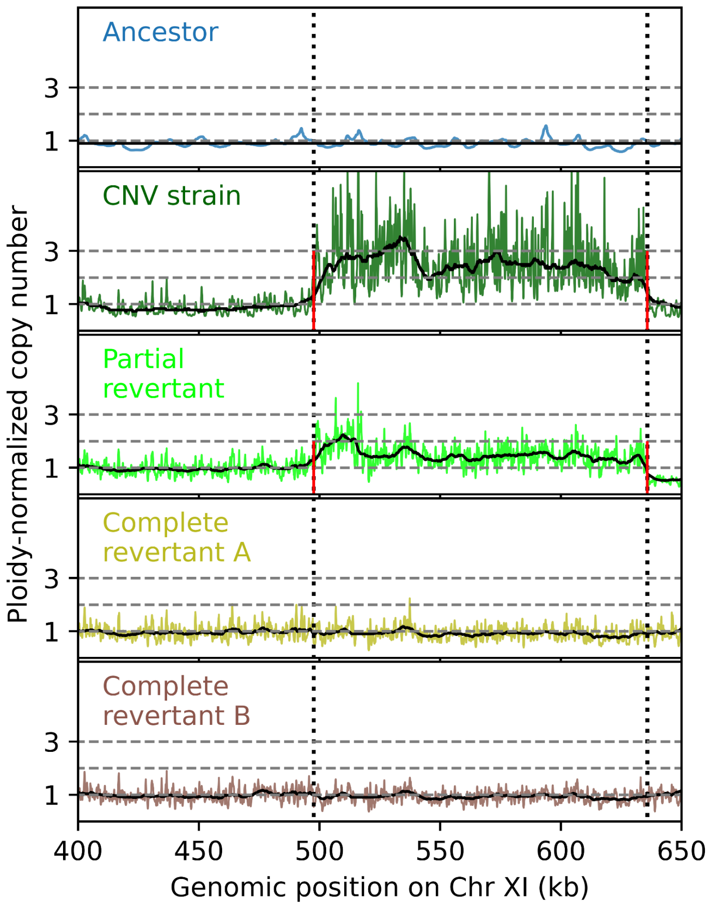


**Figure S9. Short-read whole-genome sequencing of segmental CNV strain *G_4* and derived revertants.** Sequencing read depth for each nucleotide normalized to the genome-wide mean, and averaged over all copies of the chromosome (1 for haploids, 2 for diploids), is the ploidy-normalized copy number (PNCN). Split reads (in red) indicate CNV breakpoints. CNV boundaries are marked by vertical dotted black lines; horizontal dashed grey lines indicate PNCN of 1, 2 and 3 for visual comparison. Solid black lines indicate median read depth over rolling 50 kb windows. All strains except the ancestor are diploid. We compared the CNV strain *G_4* to its ancestor, a partial revertant, and 14 complete revertants (two are shown here).

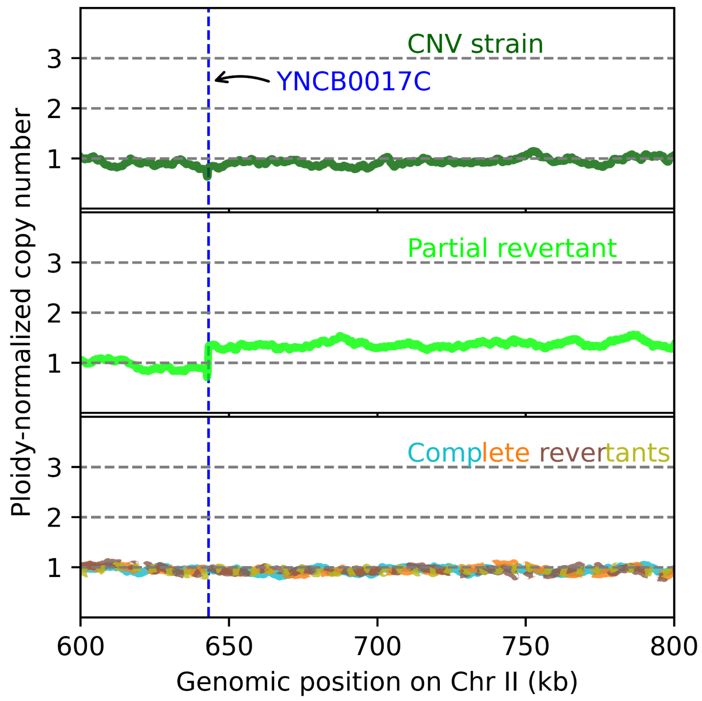


**Figure S10. Long-read sequencing showing ploidy-normalized copy number across a part of chromosome II for the segmental CNV strain *G_4* and derived revertants.** Sequencing read depth for each nucleotide normalized to the genome-wide average, and averaged over two copies of the chromosome for diploids, is the ploidy-normalized copy number (PNCN). Horizontal dashed grey lines indicate PNCN of 1, 2 and 3 for visual comparison. The vertical dashed blue line indicates the position of the *YNCB0017C* gene located at the boundary of the region translocated to the neochromosome. Shown here are the segmental CNV that underwent reversion (*G_4*), a partial revertant, and four independently evolved complete revertants (overlaid) – all diploids.

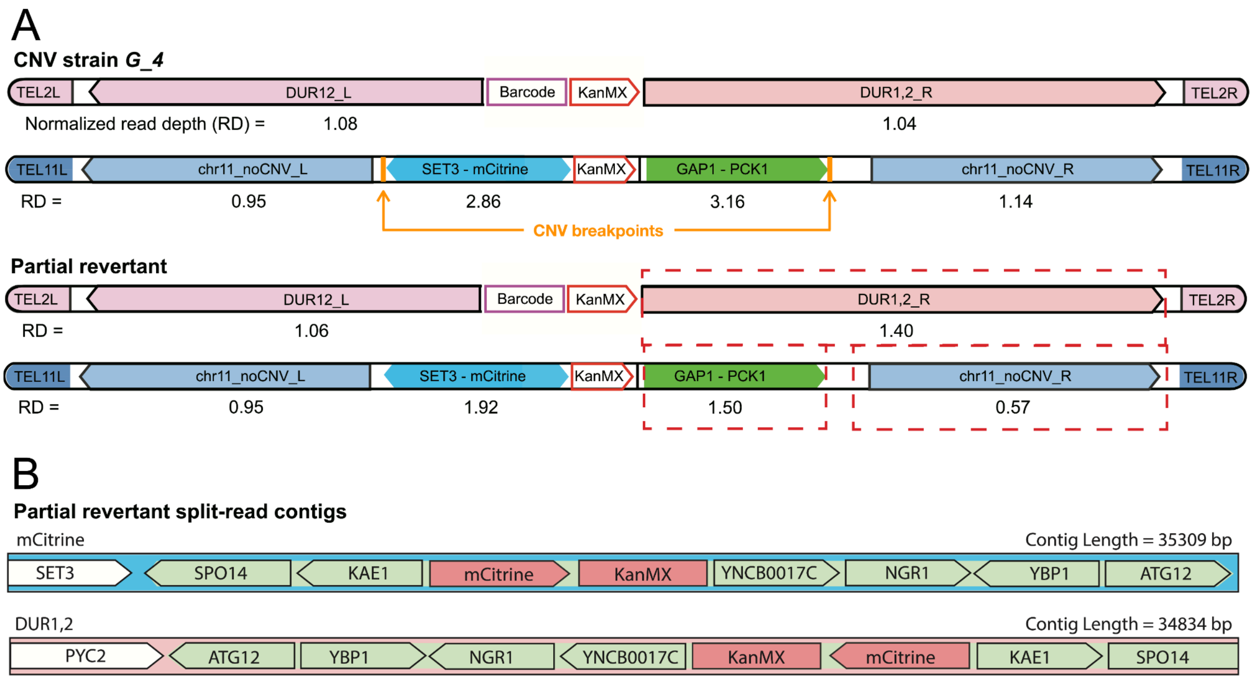


**Figure S11. De novo genome assembly and ploidy-normalized copy number reveal mechanism of partial reversion. A)** Sequencing read-depth of different parts of the amplified region relative to the genome-wide average, and then normalized by ploidy. **B)** Two contigs obtained by de novo genome assembly, which span both chromosomes XI and II.

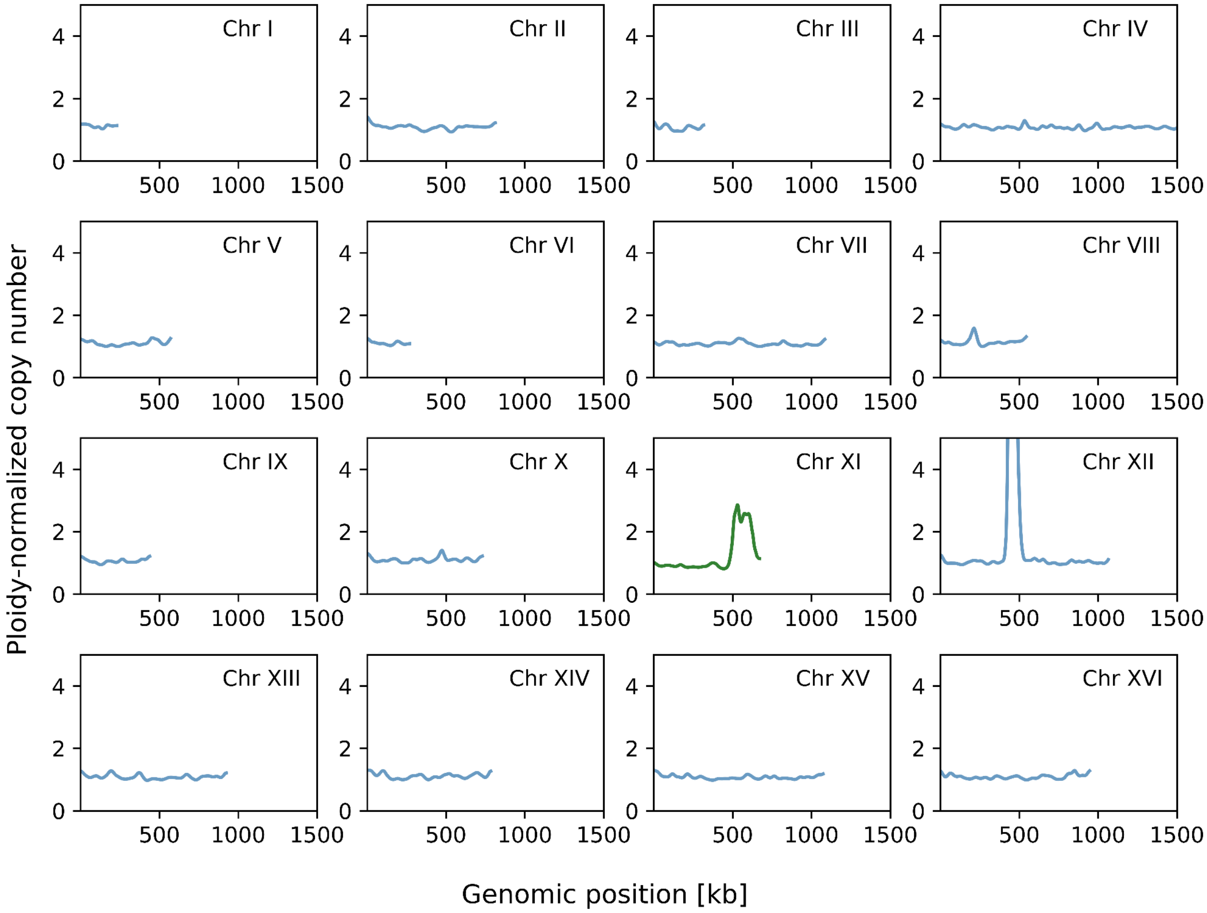


**Figure S12. Ploidy-normalized copy number across the entire length of all 16 chromosomes in the CNV strain *G_4*.** To enable direct length comparison, we used a uniform X-axis scale across all chromosomes. The CNV is present on chromosome XI, shown in green. The large peak on chromosome XII corresponds to the multicopy rDNA locus, which is only present at two copies in the reference genome, and serves as a positive control for read-depth based CNV detection.

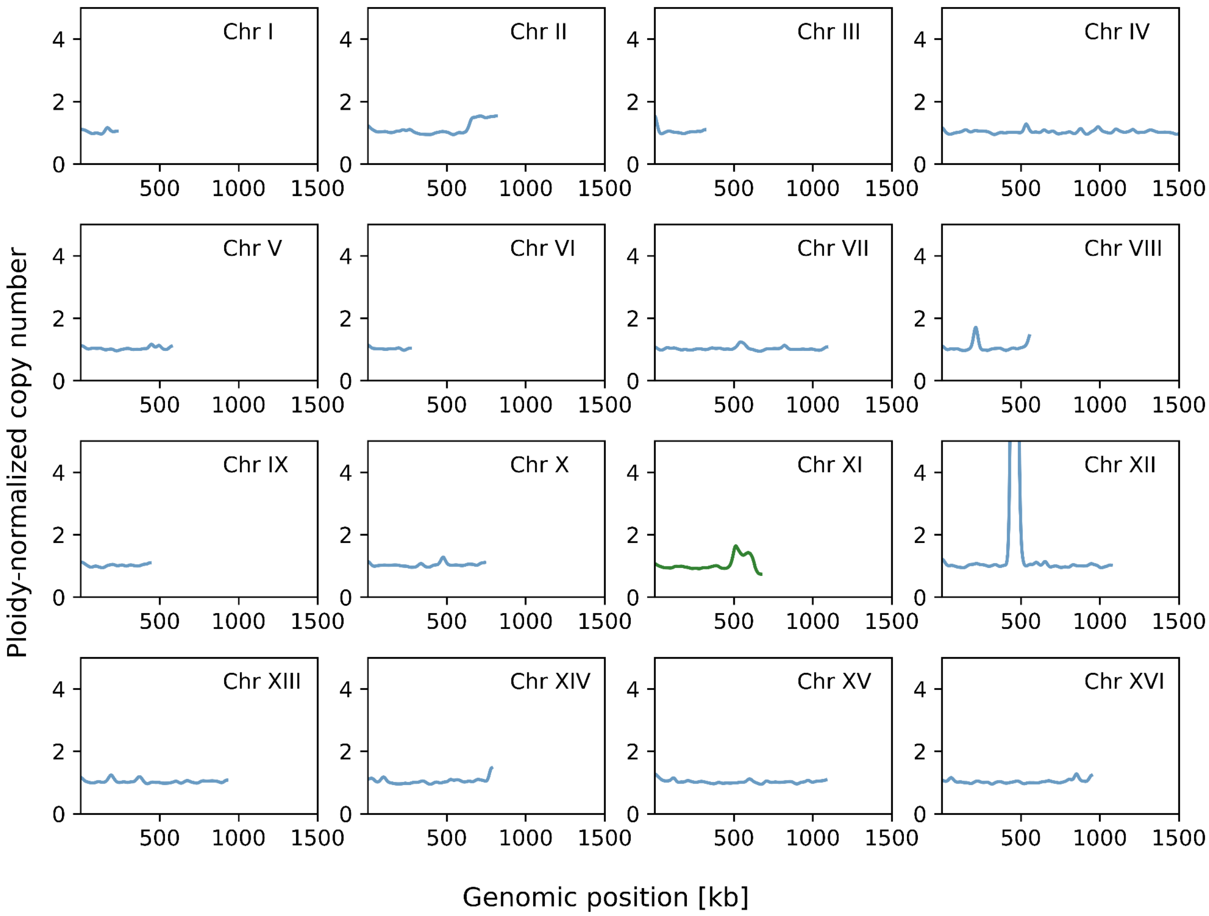


**Figure S13. Ploidy-normalized copy number across the entire length of all 16 chromosomes in the partial revertant strain.** To enable direct length comparison, we used a uniform X-axis scale across all chromosomes. The partially-reverted CNV is present on chromosome XI, shown in green. The right-hand portion of chromosome II with increased copy number corresponds to the region translocated to the neochromosome. The large peak on chromosome XII corresponds to the multicopy rDNA locus, which is only present at two copies in the reference genome, and serves as a positive control for read-depth based CNV detection.

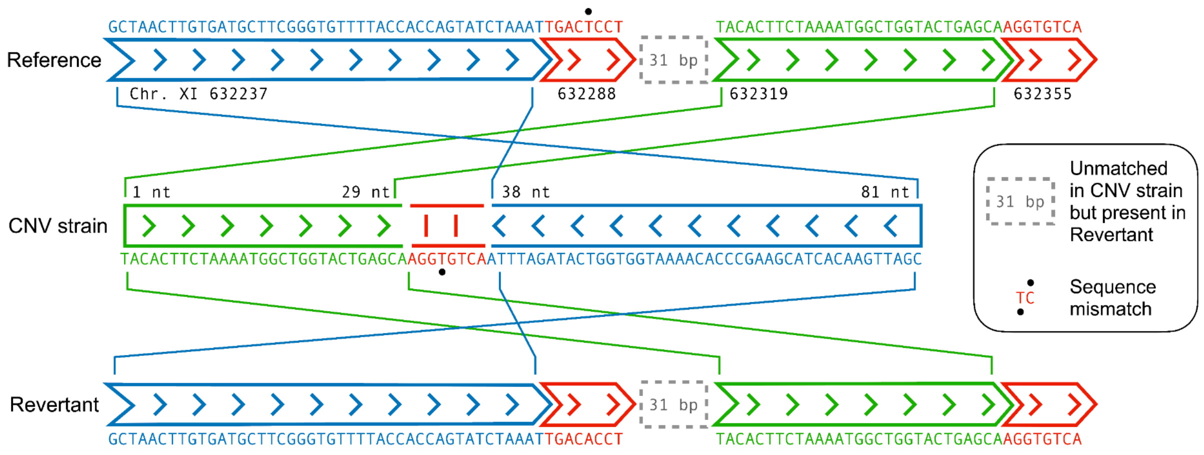


**Figure S14.** The segmental amplification, bearing an inversion in the parent CNV strain *G_4*, returns to the identical ancestral state in all 14 complete revertants (right-hand breakpoint of one example is shown here).
