# Supplementary material for: Segmental copy number amplifications are more stable than aneuploidies in the absence of selection
Source: Mol Biol Evol. 2026 Apr 11;43(4):msag095. doi: 10.1093/molbev/msag095 (PMC13107562; doi:10.1093/molbev/msag095)
Supplement: msag095_Supplementary_Data [file msag095_supplementary_data.zip › De_et_al_revision2_Supplementary_Tables_Methods.pdf]

Supplementary Tables

Table 1. **CNV strains used in this study.** Summarized here are the different parameters by which the CNVs differed: the gene(s) under selection, copy number relative to the ancestor, size of the amplified region, molecular mechanism underlying CNV formation, number of elapsed generations before the lineage was isolated and the selection condition under which they arose.

Strain	Selected gene	Copy number	Size (kb)	Formation mechanism	Generation	Nutrient limitation
G_1	*GAP1*	4	33	ODIRA, Transposons	250	Glutamine
G_2	*GAP1*	3	191	ODIRA	250	Glutamine
G_3	*GAP1*	3	174	Segmental aneuploidy	250	Glutamine
G_4	*GAP1*	3	135	ODIRA	70	Glutamine
G_5	*GAP1*	3	62	Transposons	70	Glutamine
G_6	*GAP1*	3	45	ODIRA, NHR	70	Glutamine
G_7	*GAP1*	3	16	ODIRA	62	Glutamine
G_8	*GAP1*	2	16	ODIRA	62	Glutamine
G_9	*GAP1*	2	6	Transposons	70	Glutamine
M_10	*MEP2*	3	6	HR	265	Glutamine, Ammonium
P_11	*PUT4*	3	20	ODIRA, Transposons	88	Glutamine, Proline
GM_12	*GAP1*<br>*MEP2*	2<br>2	667<br>784	Chr XI aneuploidy<br>Chr XIV aneuploidy	177	Glutamine, Ammonium
GM_13	*GAP1*<br>*MEP2*	2<br>2	667<br>784	Chr XI aneuploidy<br>Chr XIV aneuploidy	177	Glutamine, Ammonium
GM_14	*GAP1*<br>*MEP2*	2<br>2	667<br>784	Chr XI aneuploidy<br>Chr XIV aneuploidy	177	Glutamine, Ammonium
GM_15	*GAP1*<br>*MEP2*	2<br>2	667<br>784	Chr XI aneuploidy<br>Chr XIV aneuploidy	147	Glutamine, Ammonium

Table 2. **CNV strain nomenclature and origin.** Lab collection ID, and publications which reported the experiments from which each strain originated.

Name of strain in the paper	Lab collection ID	Strain origin
G_1	DGY1741	Lauer et al. (2018)
G_2	DGY1743	Lauer et al. (2018)
G_3	DGY1744	Lauer et al. (2018)
G_4	DGY1886	Lauer et al. (2018)
G_5	DGY1917	Lauer et al. (2018)
G_6	DGY1883	Lauer et al. (2018)
G_7	DGY1988	Lauer et al. (2018)
G_8	DGY1976	Lauer et al. (2018)
G_9	DGY1920	Lauer et al. (2018)
M_10	DGY2702	Abdul-Rahman et al. (in prep)
P_11	DGY2769	Abdul-Rahman et al. (in prep)
GM_12	DGY2748	Abdul-Rahman et al. (in prep)
GM_13	DGY2750	Abdul-Rahman et al. (in prep)
GM_14	DGY2751	Abdul-Rahman et al. (in prep)
GM_15	DGY2755	Abdul-Rahman et al. (in prep)

Table 3. **Unique barcodes identifying each CNV lineage and revertants descended from them.** Each sequence was obtained from whole genome sequencing data by searching for the ancestral barcode template. Variable sequence regions in each barcode are highlighted.

Ancestral barcode template (38 bp): GGTACCNNNNNAANNNNNAANNNNNTTNNNNNATAACT		
**Sequence verified barcode**	**CNV strain**	**Revertant strain**
GGTACCCGTAAAAAAGTAAAATTTTTTTAAAATATAACT<br>GGTACCCGTAAAAAAGTAAAATTTTTTTAAAATATAACT<br>GGTACCCGTAAAAAAGTAAAATTTTTTTAAAATATAACT<br>GGTACCCGTAAAAAAGTAAAATTTTTTTAAAATATAACT<br>GGTACCCGTAAAAAAGTAAAATTTTTTTAAAATATAACT<br>GGTACCCGTAAAAAAGTAAAATTTTTTTAAAATATAACT<br>GGTACCCGTAAAAAAGTAAAATTTTTTTAAAATATAACT<br>GGTACCCGTAAAAAAGTAAAATTTTTTTAAAATATAACT<br>GGTACCCGTAAAAAAGTAAAATTTTTTTAAAATATAACT<br>GGTACCCGTAAAAAAGTAAAATTTTTTTAAAATATAACT<br>GGTACCCGTAAAAAAGTAAAATTTTTTTAAAATATAACT<br>GGTACCCGTAAAAAAGTAAAATTTTTTTAAAATATAACT<br>GGTACCCGTAAAAAAGTAAAATTTTTTTAAAATATAACT<br>GGTACCCGTAAAAAAGTAAAATTTTTTTAAAATATAACT<br>GGTACCCGTAAAAAAGTAAAATTTTTTTAAAATATAACT	*G_4*	DGY3286<br>DGY3287<br>DGY3288<br>DGY3289<br>DGY3410<br>DGY3411<br>DGY3412<br>DGY3413<br>DGY3414<br>DGY3415<br>DGY3416<br>DGY3417<br>DGY3418<br>DGY3419<br>DGY3420
GGTACCTTTACAACAATTAATAGCATTATTTTATAACT	*G_5*	No CNV reversion
GGTACCCTCTGAATGCGCAAGTCTCTTTCGGGATAACT	*G_6*	No CNV reversion
GGTACCCCAAAAACAGTTAATAACATTTGAGTATAACT	*G_7*	No CNV reversion
GGTACCCTTCAAAAAATTAAGATCCTTCATTAATAACT	*G_8*	No CNV reversion
GGTACCAATGTAAGATTTAACATCCTTTTCGCATAACT	*G_9*	No CNV reversion
GGTACCAAGGAAAGCTCCAAGTGAGTTCGAAGATAACT	*M_10*	No CNV reversion
GGTACCCACATAAATGATAAAGTTGTTCGCTCATAACT	*P_11*	No CNV reversion
GGTACCTCATGAAGAAGCAAAGGGTTTAAGGAATAACT<br>GGTACCTCATGAAGAAGCAAAGGGTTTAAGGAATAACT<br>GGTACCTCATGAAGAAGCAAAGGGTTTAAGGAATAACT<br>GGTACCTCATGAAGAAGCAAAGGGTTTAAGGAATAACT<br>GGTACCTCATGAAGAAGCAAAGGGTTTAAGGAATAACT<br>GGTACCTCATGAAGAAGCAAAGGGTTTAAGGAATAACT<br>GGTACCTCATGAAGAAGCAAAGGGTTTAAGGAATAACT<br>GGTACCTCATGAAGAAGCAAAGGGTTTAAGGAATAACT<br>GGTACCTCATGAAGAAGCAAAGGGTTTAAGGAATAACT<br>GGTACCTCATGAAGAAGCAAAGGGTTTAAGGAATAACT	*GM_12*	DGY3290<br>DGY3421<br>DGY3422<br>DGY3423<br>DGY3424<br>DGY3425<br>DGY3426<br>DGY3427<br>DGY3428<br>DGY3429

GGTACCTTGAAAAATGGTAAAACAATTGTTAGATAACT<br>GGTACCTTGAAAAATGGTAAAACAATTGTTAGATAACT<br>GGTACCTTGAAAAATGGTAAAACAATTGTTAGATAACT<br>GGTACCTTGAAAAATGGTAAAACAATTGTTAGATAACT<br>GGTACCTTGAAAAATGGTAAAACAATTGTTAGATAACT<br>GGTACCTTGAAAAATGGTAAAACAATTGTTAGATAACT<br>GGTACCTTGAAAAATGGTAAAACAATTGTTAGATAACT	*GM_13*	DGY3291<br>DGY3430<br>DGY3431<br>DGY3432<br>DGY3433<br>DGY3434<br>DGY3435
GGTACCTAGTCAATATATAAAGATATTTGTGCATAACT<br>GGTACCTAGTCAATATATAAAGATATTTGTGCATAACT<br>GGTACCTAGTCAATATATAAAGATATTTGTGCATAACT<br>GGTACCTAGTCAATATATAAAGATATTTGTGCATAACT<br>GGTACCTAGTCAATATATAAAGATATTTGTGCATAACT<br>GGTACCTAGTCAATATATAAAGATATTTGTGCATAACT<br>GGTACCTAGTCAATATATAAAGATATTTGTGCATAACT<br>GGTACCTAGTCAATATATAAAGATATTTGTGCATAACT<br>GGTACCTAGTCAATATATAAAGATATTTGTGCATAACT<br>GGTACCTAGTCAATATATAAAGATATTTGTGCATAACT<br>GGTACCTAGTCAATATATAAAGATATTTGTGCATAACT	*GM_14*	DGY3293<br>DGY3439<br>DGY3440<br>DGY3441<br>DGY3442<br>DGY3443<br>DGY3444<br>DGY3445<br>DGY3446<br>DGY3447<br>DGY3448
GGTACCATCTTAAGTATCAACCACTTTCCGGCATAACT<br>GGTACCATCTTAAGTATCAACCACTTTCCGGCATAACT<br>GGTACCATCTTAAGTATCAACCACTTTCCGGCATAACT<br>GGTACCATCTTAAGTATCAACCACTTTCCGGCATAACT<br>GGTACCATCTTAAGTATCAACCACTTTCCGGCATAACT<br>GGTACCATCTTAAGTATCAACCACTTTCCGGCATAACT<br>GGTACCATCTTAAGTATCAACCACTTTCCGGCATAACT<br>GGTACCATCTTAAGTATCAACCACTTTCCGGCATAACT<br>GGTACCATCTTAAGTATCAACCACTTTCCGGCATAACT<br>GGTACCATCTTAAGTATCAACCACTTTCCGGCATAACT<br>GGTACCATCTTAAGTATCAACCACTTTCCGGCATAACT<br>GGTACCATCTTAAGTATCAACCACTTTCCGGCATAACT<br>GGTACCATCTTAAGTATCAACCACTTTCCGGCATAACT	*GM_15*	DGY3294<br>DGY3295<br>DGY3449<br>DGY3450<br>DGY3451<br>DGY3452<br>DGY3453<br>DGY3454<br>DGY3455<br>DGY3456<br>DGY3457<br>DGY3458<br>DGY3459

Table 4. Marginal prior distributions of the evolutionary parameters. The overall prior distribution is the hypercube defined by these uniform marginal distributions.

Parameter	Description	Prior
s	Selection coefficients of CNV reversion	$log_{10}(s) \sim U[-3, -1]$
δ	CNV reversion rate	$log_{10}(\delta) \sim U[-5, -2]$
φ	Initial frequency of reversed CNV cells	$log_{10}(\varphi) \sim U[-5, -0.5]$

Table 5. **Confirmed SNVs across all CNV strains and revertants.** For each SNV, we record the genomic position, the original base and the new base to which it changed, the percentage of reads which underwent the base change, the gene or intergenic region where the SNV is located, and whether the revertant underwent partial (lost only Chr XIV) or complete (lost both Chr XI and Chr XIV) reversion. We did not detect any high-confidence SNVs in any of the 15 CNV strains used in the study.

CNV Strain	Revertant strain	Chr	Position (bp)	Original base	New base	Percentage of reads	Gene or intergenic region	Reversion type
GM_12	GM_12_Rev1	VII	245951	A	G	94% of 140	FLC3	Complete
	GM_12_Rev1	XIII	712911	C	T	95% of 169	ERG8	Complete
	GM_12_Rev2	I	54719	G	A	95% of 138	GEM1	Complete
	GM_12_Rev6	IV	675218	C	T	94% of 63	YDR109C	Complete
	GM_12_Rev10	XV	626108	C	A	96% of 77	SLP1	Partial
GM_13	GM_13_Rev1	X	536002	C	G	96% of 56	Between BFA1 and KCH1	Complete
	GM_13_Rev2	XI	669969	G	A	92% of 187	Near GEX2	Complete
	GM_13_Rev6	V	530182	A	T	96% of 117	RAD3	Complete
GM_14	GM_14_Rev1	XIII	160549	G	A	94% of 101	CMP2	Complete
	GM_14_Rev1	XIII	160563	A	G	97% of 106	CMP2	Complete
	GM_14_Rev2	III	226561	T	C	94% of 89	TVS1 (YCR061W)	Complete
	GM_14_Rev10	XVI	604956	C	A	100% of 96	SDD4 (YPR022C)	Partial
GM_15	GM_15_Rev1	X	517755	A	T	98% of 127	Between POL32 and IMT3	Complete
	GM_15_Rev3	VIII	170137	G	A	97% of 153	SLT2	Complete
	GM_15_Rev6	IV	1171363	C	T	96% of 142	PAL1	Partial
	GM_15_Rev6	V	490478	C	T	93% of 184	Between tE(UUC)E3 and YER158C	Partial
	GM_15_Rev7	XI	71825	A	T	93% of 117	PEX1	Complete
	GM_15_Rev8	VIII	240308	T	C	98% of 135	ERG7	Complete
	GM_15_Rev8	XV	410348	C	T	95% of 105	Between snR62 and WHI2	Complete
	GM_15_Rev12	IV	645268	C	G	95% of 129	Between tQ(UUG)D2 and YDR098C-B	Complete

Table 6. Strain nomenclature for all revertants. Names of all revertant as used in the paper, and their corresponding lab collection ID.

Revertant	Lab collection ID		Revertant	Lab collection ID
G_4_Rev1	DGY3286		GM_13_Rev4	DGY3432
G_4_Rev2	DGY3287		GM_13_Rev5	DGY3433
G_4_Rev3	DGY3288		GM_13_Rev6	DGY3434
G_4_Rev4	DGY3289		GM_13_Rev7	DGY3435
G_4_Rev5	DGY3410		GM_14_Rev1	DGY3293
G_4_Rev6	DGY3411		GM_14_Rev2	DGY3439
G_4_Rev7	DGY3412		GM_14_Rev3	DGY3440
G_4_Rev8	DGY3413		GM_14_Rev4	DGY3441
G_4_Rev9	DGY3414		GM_14_Rev5	DGY3442
G_4_Rev10	DGY3415		GM_14_Rev6	DGY3443
G_4_Rev11	DGY3416		GM_14_Rev7	DGY3444
G_4_Rev12	DGY3417		GM_14_Rev8	DGY3445
G_4_Rev13	DGY3418		GM_14_Rev9	DGY3446
G_4_Rev14	DGY3419		GM_14_Rev10	DGY3447
G_4_Rev15	DGY3420		GM_14_Rev11	DGY3448
GM_12_Rev1	DGY3290		GM_15_Rev1	DGY3294
GM_12_Rev2	DGY3421		GM_15_Rev2	DGY3295
GM_12_Rev3	DGY3422		GM_15_Rev3	DGY3449
GM_12_Rev4	DGY3423		GM_15_Rev4	DGY3450
GM_12_Rev5	DGY3424		GM_15_Rev5	DGY3451
GM_12_Rev6	DGY3425		GM_15_Rev6	DGY3452
GM_12_Rev7	DGY3426		GM_15_Rev7	DGY3453
GM_12_Rev8	DGY3427		GM_15_Rev8	DGY3454
GM_12_Rev9	DGY3428		GM_15_Rev9	DGY3455
GM_12_Rev10	DGY3429		GM_15_Rev10	DGY3456
GM_13_Rev1	DGY3291		GM_15_Rev11	DGY3457
GM_13_Rev2	DGY3430		GM_15_Rev12	DGY3458
GM_13_Rev3	DGY3431		GM_15_Rev13	DGY3459

Supplementary Methods

Evolutionary model and simulation-based inference

Evolutionary model (without epistasis). The following presents a model for the evolution of *GAP1* CNV reversion. We follow the frequencies of two genotypes over time: X_{G_1}, the frequency of genotype G_1, representing single-copy *GAP1*; and X_{G_2}, the frequency of genotype G_2, representing two copies of *GAP1*.

The model for *MEP2* CNV reversion is similar, with M (for *MEP2*) replacing G (for *GAP1*).

Initially, the genotype frequencies are:

$$X_{G_1}^{0} = \varphi_G, \; X_{G_2}^{0} = 1 - \varphi_G .$$

where φ_G is the initial frequency of genotype G_1 and is a model parameter.

The change in frequencies due to mutation is given by

$$X_{G_2}' = (1 - \delta_G)X_{G_2}^{t}, \; X_{G_1}' = X_{G_1}^{t} + \delta_G X_{G_2}^{t} .$$

where X_g' is the frequency of genotype g after mutation.

The change in frequencies due to selection is given by

$$\bar{w} = w_{G_1}X_{G_1} + w_{G_2}X_{G_2} = (1 + s)X_{G_!} + X_{G_2} ,$$

$$X_{G_2}^{*} = \frac{X_{G_2}'}{\bar{w}}, \; X_{G_1}^{*} = \frac{X_{G_1}'}{\bar{w}}$$

where w_g is the fitness of genotype g, and $\bar{w}$ is the population mean fitness. X_g^{*} represents the frequency of genotype g after selection.

The change in frequencies due to random genetic drift is given by

$$n_{G_1} = Binomial(N_e, X_{G_1}^{*}),\ n_{G_2} = N_e - N_{G_1}$$

where n_g is the number of cells with genotype g.

The frequencies in the next generation are then given by

$$X_{G_1}^{t+1} = \frac{n_{G_1}}{N_e},\ X_{G_2}^{t+1} = \frac{n_{G_2}}{N_e}$$

Overall, we estimate three model parameters: s_G, δ_G, φ_G. The effective population size is constant at $N_e = 1.9 \times 10^6$ (see details below).

The evolutionary model for *MEP2* CNV reversion is similar, with s_M, δ_M, φ_M substituting s_G, δ_G, φ_G, and X_{M_1}, X_{M_2} substituting X_{G_1}, X_{G_2}.

Effective population size. Populations begin each full growth cycle at $N_0 = 6.25 \times 10^5$ cells and duplicate six times before back-dilution to N_0. Using the harmonic mean [Gillespie 2004] for calculating the effective population size, we have

$$N_e = \frac{6}{\frac{1}{6.25\times10^5} + \frac{1}{2\times6.25\times10^5} + \ldots \frac{1}{2^5\times6.25\times10^5}}$$

The denominator can be calculated using a geometric sum, yielding

$$\frac{1}{6.25\times10^5} + \frac{1}{2\times6.25\times10^5} + \ldots + \frac{1}{2^5\times6.25\times10^5} = \frac{\frac{1}{6.25\times10^5}(0.5^6-1)}{0.5-1}$$

Leading to

$$N_e = \frac{6}{\frac{\frac{1}{6.25\times10^5}(0.5^6-1)}{0.5-1}} \approx 1.9 \times 10^6$$

Evolutionary model with epistasis. The following describes a model for evolution of *GAP1* and *MEP2* CNV reversions with epistasis between the two CNVs (Figure S8_3). In this model, we consider four genotypes: G_2M_2, cells with both *GAP1* CNV and *MEP2* CNV (i.e., at least one *MEP2* duplication); G_1M_2, cells with a single copy of *GAP1* and a *MEP2* CNV; G_2M_1, cells with a *GAP1* CNV and two copies of MEP2; and G_1M_1, cells with single copies of both *GAP1* and *MEP2*. The frequency of genotype g at time t is denoted by X_g^t.

Each genotype has a distinct fitness, $w_g = 1 + s_g$, with the fitness of the double CNV genotype G_2M_2 fixed as 1 without loss of generality. Each of the three mutant genotypes starts with a distinct initial frequency, denoted by φ_g; and the reversion rate of CNV c is denoted by δ_c, with $c = M$ for *MEP2* and $c = G$ for *GAP1*. The rate of a second reversion j is distinct and dependent on the first reversion i, denoted by $\delta_{j|i}$. This model therefore has ten parameters: $s_{G_1M_2}, s_{G_2M_1}, s_{G_1M_1}, \delta_G, \delta_M, \delta_{G|M}, \delta_{M|G}, \varphi_{G_1M_2}, \varphi_{G_2M_1}, \varphi_{G_1M_1}$.

Assuming that CNV reversion rates are independent of the genotype, and that there is no epistasis between CNV reversions, we can calculate the values of the four parameters that are unique to the model with epistasis from the six parameters of the model without epistasis: $1 + s_{G_1M_1} = (1 + s_{G_1M_2})(1 + s_{G_2M_1})$; $\delta_{M|G} = \delta_M$, $\delta_{G|M} = \delta_G$; $\varphi_{GM} = \varphi_G \times \varphi_M$.

Initially, the genotype frequencies are:

$$X_{G_1M_1}^0 = \varphi_{G_1M_1},\ X_{G_2M_1}^0 = \varphi_{G_2M_1},\ X_{G_1M_2}^0 = \varphi_{G_1M_2},$$

$$X_{G_2M_2}^0 = 1 - \varphi_{G_1M_1} - \varphi_{G_1M_2} - \varphi_{G_2M_1}.$$

where φ_G is the initial proportion of genotype G_1, and is a model parameter.

The change in frequencies due to mutation is given by

$$X_{G_2M_2}' = (1 - \delta_G - \delta_M)X_{G_2M_2}^t,$$

$$X_{G_1M_2}' = (1 - \delta_M)X_{G_1M_2}^t + \delta_G X_{G_2M_2}^t$$

$$X_{G_2M_1}' = (1 - \delta_G)X_{G_2M_1}^t + \delta_M X_{G_2M_2}^t$$

$$X_{G_1M_1}' = X_{G_1M_1}^t + \delta_G X_{G_2M_1}^t + \delta_M X_{G_1M_2}^t$$

where X_g' is the frequency of genotype g after mutation.

The change in frequencies due to selection is given by

$$\bar{w} = \Sigma_g [w_g X_g]\ , X_g^* = \frac{X_g'}{\bar{w}}$$

where w_g is the fitness of genotype g, and $\bar{w}$ is the population mean fitness. X_g^* represents the frequency of genotype g after selection.

The change in frequencies due to random genetic drift is given by

$$(n_{G_1M_1}, n_{G_1M_2}, n_{G_2M_1}, n_{G_2M_2}) = Multinomial(N_e, (X_{G_1M_1}^*, X_{G_1M_2}^*, X_{G_2M_1}^*, X_{G_2M_2}^*))$$

where n_g is the number of cells with genotype g.

The frequencies in the next generation are then given by

$$X_g^{t+1} = \frac{n_g}{N_e}$$

Predictive checks for the model with epistasis. To perform a predictive check, we inferred six model parameters using the model without epistasis for each of the replicates, calculated the value of the additional four parameters assuming independence of CNV reversions, simulated using these parameter estimates in the model with epistasis, and compared the simulation results to the empirical data.
These predictions aligned well with the empirical data. Therefore, we concluded that the model without epistasis is sufficient and did not use the model with epistasis for parameter inference in the main text. In the future, the model with epistasis could be used to study results of experiments with epistasis between two-loci.
